# Crystal Structure of H227A Mutant of Arginine Kinase in *Daphnia magna* Suggests the Importance of Its Stability

**DOI:** 10.3390/molecules27030884

**Published:** 2022-01-28

**Authors:** Da Som Kim, Kiyoung Jang, Wan Seo Kim, Moonhee Ryu, Jung Hee Park, Yong Ju Kim

**Affiliations:** 1 Division of Biotechnology, College of Environmental & Bioresources Sciences, Jeonbuk National University, Iksan 54596, Korea; jboa@jbnu.ac.kr (D.S.K.); wanseo0326@jbnu.ac.kr (W.S.K.); ryumh@jbnu.ac.kr (M.R.); 2 Department of Lifestyle Medicine, College of Environmental and Bioresource Sciences, Jeonbuk National University, Iksan 54596, Korea; kiyoung64@jbnu.ac.kr; 3 Advanced Institute of Environment and Bioscience, College of Environmental & Bioresources Sciences, Jeonbuk National University, Iksan 54596, Korea; 4 Department of Oriental Medicine Resources, College of Environmental and Bioresource Sciences, Jeonbuk National University, Iksan 54596, Korea

**Keywords:** arginine kinase, protein crystal, X-ray diffraction, circular dichroism spectroscopy, structural stability

## Abstract

Arginine kinase (AK) plays a crucial role in the survival of *Daphnia magna*, a water flea and a common planktonic invertebrate sensitive to water pollution, owing to the production of bioenergy. AK from *D. magna* (*Dm*AK) has four highly conserved histidine residues, namely, H90, H227, H284, and H315 in the amino acid sequence. In contrast to *Dm*AK WT (wild type), the enzyme activity of the H227A mutant decreases by 18%. To identify the structure-function relationship of this H227A mutant enzyme, the crystal 3D X-ray structure has been determined and an unfolding assay using anilino-1-naphthalenesulfonic acid (ANS) fluorescence has been undertaken. The results revealed that when compared to the *Dm*AK WT, the hydrogen bonding between H227 and A135 was broken in the H227A crystal structure. This suggests that H227 residue, closed to the arginine binding site, plays an important role in maintaining the structural stability and maximizing the enzyme activity through hydrogen bonding with the backbone oxygen of A135.

## 1. Introduction

*Daphnia magna*, a model organism of water fleas and a common planktonic invertebrate [1], is widely used as an experimental subject because it is highly sensitive to toxicants and has a fast-breeding cycle [2]. Some studies on the relationship between cyanobacteria and zooplankton have shown that cyanobacteria suppress zooplankton growth, reproduction, and survival by producing the toxin microcystin [3]. However, recent studies have revealed that *Daphnia* clones are adapted to environments containing toxic cyanobacteria and are resistant to algal abundance [4,5]. Based on these results, researchers suggested that *D. magna* resists toxic cyanobacteria by energy coupling through the upregulation of arginine kinase (AK) [6].

AK belongs to the phosphagen kinase family and acts as an energy modulator when bioenergy is required in invertebrate cells, by reversibly converting phosphoarginine and ADP to L-arginine and ATP [7].

(1) $$\mathrm{Arginine}\;\mathrm{phosphate}+\mathrm{Mg}\cdot {\mathrm{ADP}}^{-}+H^{+}\overset{Arginine\;kinase}{↔}\;\mathrm{phosphate}+\mathrm{Mg}\cdot {\mathrm{ATP}}^{2-}$$

Arginine is an essential amino acid for all living organisms and acts as a substrate for nitric oxide synthase in the citrulline-NO cycle and arginase in the urea cycle [8]. In addition, recent studies have been conducted on environmental stress in *Callinectes sapidus* [9], oxidative stress in *Trypanosoma cruzi* [10], cadmium acclimation in *Eriocheir sinensis* [11], abiotic stress, including heavy metals, temperature, pesticides, and herbicides in *Apis cerana* [12], and pH stress in *Patinopecten yessoensis* [13]. These studies have provided evidence that AK is an important indicator of stress response in various species.

**Figure 1:**
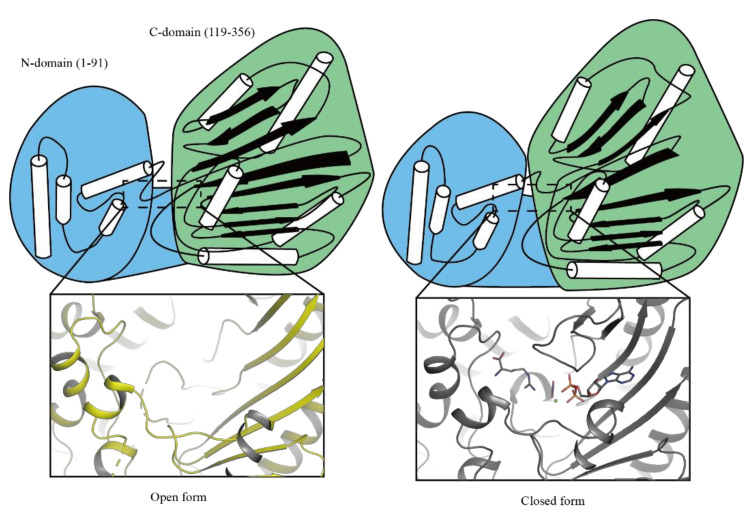
Substrate binding-dependent conformational changes in *Limulus polyphemus* AK. The structure of AK has been separated by two domains, N-domain (blue) and C-domain (green). Apoenzyme has **open form** (PDB: 3M10, yellow) and holoenzyme has **closed form** (PDB: 5J99, black) [14,15].

In structural aspects, both AK_apo_ and AK_holo_ have been shown to consist of two domains, an arginine interacting in the N-terminal domain and ATP in the C-terminal domain (Figure 1) [14,15]. The conformational changes between open and closed forms depended on substrate binding. Interestingly, AKs have highly conserved amino acid residues around the active site, as reported in previous studies [16,17,18,19,20]. Their function has been elucidated using a mutagenesis study, based on which it was suggested that the conserved amino acids have a close relationship with enzyme activity and structural stability [17,19,20,21,22,23,24]. Recently, four highly conserved histidine residues located far from the active site have been identified by Rao et. al. [25]. The study uncovered that among the highly conserved histidine residues (H90, H227, H284, and H315) located around the active site of *Dm*AK, H284 contributes to the activity through the formation of a hydrogen bonding network independent of structural stability.

In this study, we report for the first time the crystal structure and stability of the *D. magna* arginine kinase (*Dm*AK) H227A mutant, which is close to the L-arginine binding site. In the enzyme kinetics and stability assay, the H227A mutant revealed a 18% lower activity and an extremely lower structural stability than the WT. Furthermore, the results revealed that the hydrogen bonding between H227 and A135 was broken in the *Dm*AK H227A crystal structure in contrast to the *Dm*AK WT. The breakage of the hydrogen bond plays a crucial role in structural stability and could help in understanding the bioenergetic mechanism in *D. magna*.

## 2. Results and Discussions

### 2.1. Assay of Enzyme Activity and Structural Stability

**Figure 2:**
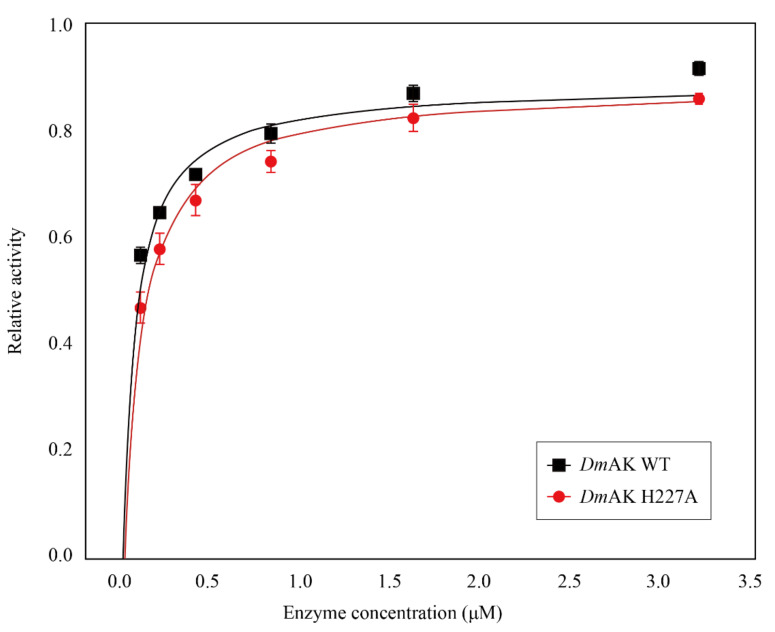
Concentation-dependent enzyme kinetics. Kinetics comparison of *Dm*AK WT (black) and H227A (red) in terms of enzyme concentration. Data on enzymatic activity were relatively evaluated.

The values of *K_m_* and *K_d_* were measured and calculated for each substrate. Enzyme kinetics of *Dm*AK WT and H227A were analyzed under optimal conditions as previously reported by Rao et. al. [25]. The results showed that the activity of *Dm*AK H227A was slightly lower than *Dm*AK WT in a concentration-dependent manner (Figure 2). Table 1 presents the determination of kinetic parameters, according to which, the *K_m_* and *K_d_* values for L-arginine and ATP had no significant difference between *Dm*AK WT and H227A. On the other hand, the catalytic efficiencies (*K*_cat_/*K_m_*) of L-arginine were 17.27 and 16.42 (mM^−1^ s^−1^) and those of ATP were 5.43 and 3.85 (mM^−1^ s^−1^) for WT and H227A, respectively. In *Dm*AK H227A, the catalytic efficiencies were 95.1% and 70.9% for L-arginine and ATP, respectively, relative to *Dm*AK WT. *V*_max_ and *K*_cat_ values for *Dm*AK H227A were lowered by 18.0% and 17.8%, respectively, relative to those of WT. It appeared that the activity reduction was not affected by chemical bonding but by structural instability. Therefore, to decipher the role of H227 accurately, the stability was tested by performing the ANS assay.

**Figure 3:**
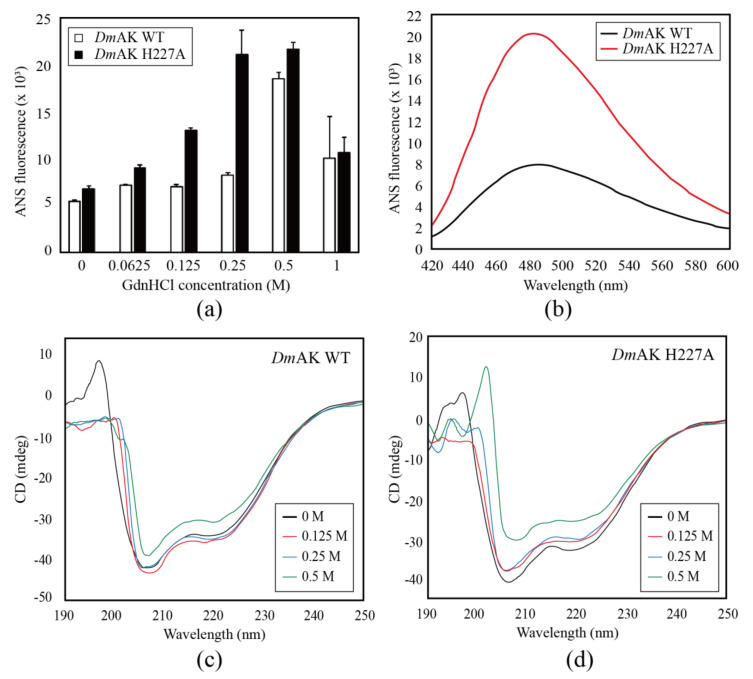
Unfolding assay with ANS fluorescence. The maximum fluorescence intensity depending on GdnHCl concentration. The greatest difference in fluorescence intensity was observed at a GdnHCl concentration of 0.25 M (**a**). The unfolding of *Dm*AK WT and H227A was analyzed based on a GdnHCl concentration of 0.25 M. Each wavelength of the maximum intensity was 480 nm for H227A and 485 nm for *Dm*AK WT (**b**). CD spectra of *Dm*AK WT (**c**) and *Dm*AK H227A (**d**) were measured from 190 nm to 260 nm with serial GdnHCl concentrations, 0 M (black), red (0.125 M), blue (0.25 M) and green (0.5 M), respectively.

For the ANS assay of *Dm*AK WT and H227A, the concentration of GdnHCl solution was varied from 0 to 1 M and applied as a protein denaturant (Figure 3a). This result implied that the H227A mutant had a more hydrophobic region than WT. The fluorescence intensities of both *Dm*AK WT and H227A increased gradually in the presence of GdnHCl in a concentration-dependent manner. Interestingly, *Dm*AK H227A presented a greater variation in the fluorescence intensity than *Dm*AK WT. In addition, the greatest difference in fluorescence intensity in both *Dm*AK WT and H227A was 2.56-fold at 0.25 M GdnHCl (Figure 3b). This observation signified that an enormous hydrophobic core exposure occurred during protein denaturation in the H227A mutant. The protein stability of *Dm*AK and H227A mutant was investigated by the ANS assay using GdnHCl as a denaturant agent by applying concentrations up to 1 M. The graphs reported in Figure 3a show the progressive increment of the ANS fluorescence up to 0.5 M GdnHCl and a decrement at 1 M, reaching values still higher than those recorded without the denaturing agent. The same behavior is ascertained for *Dm*AK WT. The determined curves are not unfolding curves but show an increment of the fluorescence signal by exposing the proteins at the specific concentrations of GdnHCl. A structural effect induced by GdnHCl was suggested, which does not seem like denaturation; indeed the sign reduced at higher GdnHCl concentrations. To better investigate the structural effect, we determine the effect of the mutation on protein stability by CD spectroscopy.

In the far-UV region of the CD spectra, the secondary structure of *Dm*AK WT and H227A was monitored from 190 nm to 250 nm. Particularly, the α-helix structure shows a strong positive maximum at 192 nm and two negative minimums at 208 and 222 nm. Comparing the CD signals, *Dm*AK H227A at 222 nm shows a steady reduction by serial GdnHCl concentrations (0, 0.125, 0.25, and 0.5 M), in contrast, *Dm*AK WT shows a decrease in CD signal over 0.25 M after a momentary increase from 0 to 0.125 M (Figure 3c,d). Additionally, the absorption intensity of *Dm*AK WT was slightly lower than H227A, approximately 2.79% of the CD signal at 222 nm, at the 0 M concentration of GdnHCl (Figure S1, Supplementary Materials). It shows that the α-helix structure of WT is more stable than H227A.

**Figure 4:**
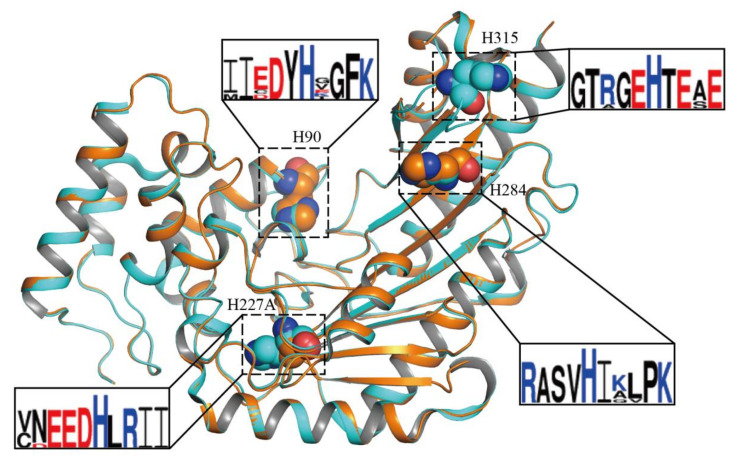
Sequence alignment of four highly conserved histidine residues and the overall structure of *Dm*AK WT and H227A. Superimposed model of *Dm*AK (PDB ID: 6KY2, cyan color) and *Dm*AK H227A (PDB ID: 7VCJ, orange color) are shown. The histidine residues are shown as spheres. Sequence logos for *Dm*AK were generated using the WebLogo3 tool [26].

All *Dm*AK have four histidine residues (H90, H227, H284, and H315; Figure 4) that were also mutated and assayed by ANS (Figure S2). Four mutants of histidine residues, except for H227A, did not show significant results. Of the four histidine residues, H227 and H284 were locationally close to the active site of *Dm*AK. In contrary to previously reported H284A mutant study, H227A mutant had an effect on structural stability but not on activity [25].

### 2.2. The Overall Structure of DmAK H227A

The *Dm*AK H227A structure belongs to the C121 space group with unit cell parameters of a = 78.146 Å, b = 58.209 Å, c = 75.412 Å, and β = 100.48°. Following the Matthews’s coefficient calculation, the solvent content and crystal volume per protein weight are 40.47% and 2.06 Å^3^ of V_M_ Da^−1^, respectively. In the final refinement at 1.75 Å, *R_work_* and *R_free_* values of *Dm*AK H227A were 0.181 and 0.224, respectively (Table 2).

The overall structure of *Dm*AK H227A had 360 amino acids, 320 water molecules, and 1 ion each of PO_4_^3−^ and NO_3_^−^. Although cocrystallization with ADP and L-arginine substrates was conducted, their presence was not detected in the *Dm*AK H227A structure. In the *Dm*AK H227A structure, the N-terminal domain (1–90) was found to consist of a series of α-helices, and the C-terminal domain (119–356) was observed to contain an eight-stranded antiparallel β-sheets connected to seven α-helices. In contrast to the *Dm*AK WT_apo_ structure (PDB ID: 6KY2) [25], eight amino acid residues (A312-E319) in *Dm*AK H227A, known as a specific loop, linked the 7th and 8th β-sheets, were not modeled [25,27]. However, the overall topology of the *Dm*AK H227A structure showed no significant difference (Figure 4). The root-mean-square deviation was estimated as 0.326 Å for Cα atoms in the superposition of *Dm*AK WT and H227A.

### 2.3. Structural Features of DmAK H227A

**Figure 5:**
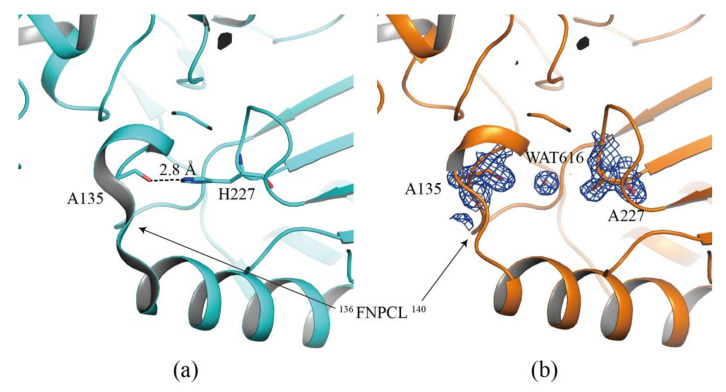
Structural comparison around the H227 residue between *Dm*AK WT (**a**) and *Dm*AK H227A (**b**). Compared to *Dm*AK WT, the hydrogen bonding was disrupted in *Dm*AK H227A. *Dm*AK WT and *Dm*AK H227A are shown in cyan and orange, respectively. The residues (A135, H227, and A227) and a water molecule are represented as stick and star model, respectively. 2*F*_o_—*F*_c_ electron density maps of A135, A227, and WAT684 of *Dm*AK H227A are represented as blue-colored meshes, each at a 1.0 σ contour.

The H227 residue was well conserved in other AKs and located in the loop connecting the 5^th^ and the 4^th^ β-sheets. The NE2 atom of this residue formed a hydrogen bond with the backbone carbonyl oxygen of A135, as previously reported for the *Dm*AK structure (Figure 5a) [25]. This hydrogen bond is commonly found in many AK structures, such as A131-H224 in *Apostichopus japonicus* [28] and P135-H227 in *Scylla paramamosain* [29], P135-H227 in *Polybetes pythagoricus* [30], P135-H227 in *Penaeus vannamei* [31] and P135-H227 in *Limulus Polyphemus* [14], in both the basal and transition states. In the case of the *Dm*AK H227A structure, the hydrogen bond destruction between A135 and H227, resulted in the replacement of one water molecule (WAT684) at the imidazole group of histidine position (Figure 5b). Moreover, the disruption of the hydrogen bond decreased the stability of the secondary structure of *Dm*AK H227A. Conversely, the hydrogen bonding between H227 and A135 in *Dm*AK WT strongly induced the formation of the secondary structure (short helix) of the adjacent amino acids (^136^FNPCL^140^, Figure 5a,b), which were highly conserved in the previously reported AK structures with a high resolution (Figure S3) [23,25,31]. This short helix slightly increased the distance between the O atom of F136 and the N atom of L140 from 3.2 Å to 3.5 Å (Figure S4), suggesting that the hydrogen bond connecting F136 and L140 had weakened. Even though the Cα spiral structure in ^136^FNPCL^140^ site is the same in structure, the H-bond becomes weaker when the distance is far away. It indicates that the Cα spiral structure in ^136^FNPCL^140^ site contains weak α-helical structure (Figure S4). This reduction in the structural stability of *Dm*AK H227A was consisted with the results of CD spectroscopy study (Figure 3c,d). Therefore, the stability of the secondary structure of the FNPCL loop might be dependent on the hydrogen bond.

## 3. Materials and Methods

### 3.1. Cloning, Expression, and Purification of DmAK WT and Histidine Mutants

*Dm*AK (GenBank accession No. AID69955.1) was chemically synthesized and amplified for single-site mutation by the standard PCR (Polymerase chain reaction) method and modified by the overlap PCR method with the primers (Table 3). On the preferential step of PCR, the chemically synthesized *Dm*AK gene was partially amplified by the pairs of primers. *Dm*AK-H90A-F and *Dm*AK-H227A-F were made by pairs of *Dm*AK-NotI-R. And *Dm*AK-H284-R and *Dm*AK-H315A-R were made by pairs of *Dm*AK-NdeI-F, respectively. These PCR products, containing mutated gene, was extracted by QIAquick Gel Extraction Kit (Hilden, Germany, QIAGEN) and utilized to second PCR step as primer with primer *Dm*AK-NdeI-F (Forward) and *Dm*AK-NotI-R, which didn’t use at first PCR. The mutated genes, after second PCR, were inserted into the pET30a expression vector (Ipswich, MA, USA, New England Biolabs) using a restriction enzyme of *Nde*I and *Not*I (Kusatsu, Japan, Takara). The recombinant plasmids of *Dm*AK histidine mutants (H90A, H227A, H284A and H315A) were induced to express in BL21(DE3) *Escherichia coli* (*E. coli*) system with 0.5 mM isopropyl β-D-1-thiogalactopyranoside (IPTG) (Haarlem, Netherlands, Duchefa Biochemie) for overnight at 20 °C and 170 rpm. The expression and purification of recombinant *Dm*AK WT and *Dm*AK histidine mutants were performed as previously reported [25]. All expressed *Dm*AK were successfully purified using HisTrap FF and HiTrap Q FF columns. The purified fractions were analyzed on 12% sodium dodecyl sulfate-polyacrylamide gel electrophoresis (SDS-PAGE) and gathered for concentration using 10-kDa Centrifugal Filters (Darmstadt, Germany, Merck) until achieving 10 mg/mL concentration (Figure S5a). Separated *Dm*AK WT and H227A were transferred to a polyvinylidene fluoride (PVDF) membrane (Chicago, IL, USA, GE Healthcare). Monoclonal anti-his (Seoul, Korea, BIOMAX) and Goat anti-mouse IgG F(ab’)2 (horseradish peroxidase-conjugated) (Farmingdale, IL, USA, ENZO) antibodies were used as primary and secondary antibodies, respectively. Signal detection was performed using the enhanced chemiluminescence (ECL) western detection kit (Chicago, IL, USA, GE Healthcare) (Figure S5b).

### 3.2. AK Activity Test

The optimal conditions of enzyme activity, pH, and temperature were applied as suggested by previous reports [17,25]. For comparison of the enzyme concentration-dependent activities, *Dm*AK WT and *Dm*AK H227A were prepared under optimized reaction conditions. The reaction mixture was composed of 10 mM L-arginine, 3 mM ATP, and 3 mM magnesium acetate in 100 mM Tris-HCl buffer (pH 8.5) (St. Louis, MO, USA, Sigma-Aldrich). The concentrated *Dm*AK WT and H227A were added into the 270 µL of the reaction mixture, and the final concentration of the enzyme was 0, 0.1, 0.2, 0.4, 0.8, 1.6, and 3.2 μM. After allowing to react for 1 min, the reaction was stopped with 2.5% trichloroacetic acid (TCA) (Seoul, Korea, SAMCHUN CHEMICALS). The mixture was treated on a 100 °C water bath for 1 min, cooled down in ice water for 1 min, and then incubated at room temperature for 5 min. For the detection of the inorganic phosphate levels, the mixture was treated with a PDR (phosphate determination reagent) solution [32]. After 1 min of reaction, sufficient color development was noted, which was then assayed at 660 nm and room temperature using a UV-Vis spectrophotometer (Männedorf, Switzerland, Tecan). All measurements were conducted more than thrice with purified *Dm*AK WT and H227A.

### 3.3. Spectroscopic Measurements

For the protein unfolding assay, 10 µM of *Dm*AK WT and H227A were to the standard buffer (pH 8.1) depending on the serial guanidine hydrochloride (GdnHCl) (Daejeon, Korea, LPS Solution) concentrations (0 M, 0.125 M, 0.25 M, 0.5 M and 1 M). After 400 µM anilino-1-naphthalenesulfonic acid (ANS) (St. Louis, MO, USA, Sigma-Aldrich) was treated, the mixture was incubated for two hours at 20 °C and incubated for another 30 min. The unfolding data were subsequently analyzed by fluorescence spectra (Männedorf, Switzerland, Tecan). The excitation was measured at 380 nm, while the emission range was 400–600 nm. At 0.25 M of Guanidine hydrochloride (GdnHCl), *Dm*AK WT and *Dm*AK histidine mutants of 10 µM concentration were added to the standard buffer (pH 8.1). The unfolding assay of *Dm*AK histidine mutants were performed in the same way as *Dm*AK WT and H227A.

CD (Circular Dichroism) spectra of *Dm*AK WT and H227A were monitored using JASCO J-1500 spectropolarimeter (Japan, Tokyo, JASCO) from 190 nm to 260 nm with 0.1 nm of data pitch. GdnHCl was added to the final concentration of 10 µM *Dm*AK WT and H227A, respectively. For measurement, each scan with serial concentrations (0 M, 0.125 M, 0.25 M and 0.5 M) of GdnHCl was conducted by three times of accumulations. The quartz cell was 1 nm path cell, and scanning speed was 100 nm/min. The optical system was corrected by blank with buffer of 10 mM Tris, pH 7.0 at room temperature.

### 3.4. Crystallization and Structural Determination of DmAK H227A

The first trial for the crystallization of *Dm*AK H227A was conducted by hanging drop vapor-diffusion method with the Grid Screen Ammonium Sulfate kit and Quik Screen kit (Aliso Viejo, CA, USA, HAMPTON RESEARCH) at 20 °C. Prior to setting up the crystallization, the protein was prepared in 10–15 mg/mL with 10 mM MgCl_2_, 0.5 mM L-arginine, 2.5 mM NaNO_3_, and 2 mM ADP. The protein sample was mixed with the reservoir solution in a 1:1 ratio. The initial crystal was obtained from the Grid Screen Ammonium Sulfate kit of C6 (2.4 M ammonium sulfate, 0.1 M BICINE, pH 9.0) and Quik Screen of D6 (1.8 M sodium/potassium phosphate, pH 8.2). The best concentration was deemed to be 1.8 M sodium/potassium phosphate (pH 8.2).

In the hanging drop method, monochronic crystals were grown and sustained for 7 days (Figure S5c). For data collection, the single crystal was frozen in 5% and 10% sucrose solution as a cryoprotectant in liquid nitrogen sequentially. The crystal was placed under a cold nitrogen gas stream at 100 K and detected by X-ray analysis. The data were collected at a wavelength of 0.979490 Å on the Pilatus3 6M detector from Beamline 11C µ-MX at Pohang Accelerator Laboratory, Pohang, Korea. To avoid excessive overflow during the detection by X-ray, 90% attenuation was allowed to obtain the diffraction data. The diffraction data set was gathered by using the XDS package software [33]. The single crystal was diffracted to 1.75 Å (Figure S5d).

The model of *Dm*AK H227A was obtained by PHASER-MR in ccp4i with *Dm*AK WT (PDB ID: 6KY2) as a template [25,34]. The *Dm*AK H227A was refined by refmac5 in ccp4i [35] and fitted by WinCoot. Final refinement was performed by phenix.refine in PHENIX software [36,37]. The values of *R_free_* and *R_work_* were 22.4% and 18.1%, respectively (Table 2). The structure was validated with the PROCHECK program in ccp4i [38] and wwPDB OneDep system [39]. The coordinate and structural factors of *Dm*AK H227A were deposited in the wwPDB with the accession code 7VCJ.

## 4. Conclusions

To date, many site-directed mutagenesis of AKs in invertebrate have been performed in highly conserved amino acids (G66, C271 and T273) located in bi-substrates binding site [17,21,40,41]. The results revealed that the mutants lost both activity and structural stability. It indicates that role of mutant is determined by closed relationship between activity and structural stability. Unfortunately, their results did not explain directly the loss of activity and structural stability at molecular level. Our study of the *Dm*AK H227A mutant suggests that the reason for the loss of structural stability in the molecular aspect, which has not been shown in the results of previous AK mutant studies.

In the crystal structure of *Dm*AK H227A, the hydrogen bond disruption between H227 and A135 resulted in the lowered structural stability of *Dm*AK H227A due to the weakness of α-helix formation of the FNPCL residues as also shown by CD signal data. These structural feature of *Dm*AK H227A is also difference with *Dm*AK H284 structure reported previously (PDB ID: 6KY3). The H284 residue might play the rearrangement of specific loop when ATP binds to *Dm*AK. The highly conserved H227 residue adjacent to the arginine binding site affected the structural stability of *Dm*AK, and the turnover (*K_cat_*) value of H227A decreased by about 17.9% compared to the wild type as shown by the enzyme kinetic results. We suggest the proposed modalities of structural stability in *Dm*AK H227A (Figure S6); (i) the mutation of H227A breaks the hydrogen bond between A135 and H227A, (ii) it might induce the weakness of secondary structure in ^136^FNPCL^140^ loop because of the increasing distance of F136 and L140, (iii) the water molecule occupied the space in which imidazole group of H227 was located. Taken together, the role of the H227 residue could contribute to the regulation of the homeostasis of cellular energy required for the survival of *D. Magna*. Therefore, our results provide a clue for the elucidation of the bioenergetic mechanism of AKs.

## Figures and Tables

**Table 1:** Comparison of the kinetic parameters of *Dm*AK WT and *Dm*AK H227A.

	*V*_max_ (μmolpimin^−1^ mg^−1^)	*K*_cat_ (s^−1^)	*K_m_^Arg^* (mM)	*K_m_^ATP^* (mM)	*K_d_^Arg^* (mM)	*K_d_^ATP^* (mM)
***Dm*** **AK WT**	318.4 ± 7.38	3.80 ± 0.09	0.22 ± 0.04	0.70 ± 0.09	2.41 ± 0.34	0.80 ± 0.10
***Dm*** **AK H227A**	261.0 ± 8.61	3.12 ± 0.10	0.19 ± 0.06	0.81 ± 0.13	2.74 ± 0.54	0.68 ± 0.11

**Table 2:** Data collection and refinement.

Data Collection	
X-ray source (Detector)	PAL 11C µ-MX (Pilatus3 6M)
Wavelength (Å)	0.979490
Space group	C121
Unit cell dimensions *a, b, c* (Å)	78.15, 58.21, 75.41
α, β, γ (°)	90.00, 100.48, 90.00
Resolution range (Å)	46.40–1.75 (1.78–1.75)
Unique reflections	37,443 (5325)
Completeness (%)	98.4 (98.0)
Multiplicity	3.4 (3.3)
<*I*/*σ(I)*>	12.1 (3.3)
*R_meas_*	0.068 (0.345)
Overall B factor from Wilson plot (Å ^2^)	13.0
**Refinement**	
Resolution range (Å)	46.4–1.75
Highest resolution shell (Å)	1.75
Completeness (%)	97.9
No. reflections	33,099 (1621)
*R_work_*/*R_free_* (%)	18.1/22.4
No. of atoms / residues	3123/360
Protein	2794
Others	329
PO_4_	5
NO_3_	4
Water	320
R.m.s. deviations	
Bonds length(Å)	0.008
Bond Angles (°)	1.038
Ramachandran plot	
Most favored (%)	97.7
Allowed (%)	2.0
Disallowed (%)	0.3

**Table 3:** Primer list for mutation of histidine residues.

Primer Name	Sequence
*Dm*AK-NdeI-F	5′-GCACTCCATATGCATCACCATCATCATCATGTGGAC-3′
*Dm*AK-H90A-F	5′-GTGATTACGCCACCGGCTTCAAG-3′
*Dm*AK-H227A-F	5′-ATGAGGAAGATGCCCTGAGAATTATC-3′
*Dm*AK-H284-R	5′-GCAGTGCAATGGCCACTGAGGCCC-3′
*Dm*AK-H315A-R	5′-CTGCTTCCGTGGCCTCACCAGCGGTTC-3′
*Dm*AK-NotI-R	5′-GCACTCGCGGCCGCTTATGCGGCTTC-3′

## Data Availability

The data presented in this study will be available upon request.

## Supplementary Materials

The following are available online, Figure S1: CD spectra of *Dm*AK WT and H227A. Figure S2: Protein unfolding assay of *Dm*AK WT and histidine mutants (H90A, H227A, H284A, H315A) in 0.25 M of Guanidine hydrochloride (GdnHCl) condition. Figure S3: The highly conserved FNPCL residues. The FNPCL residues in other AKs are represented by cartoon and sticks model. Figure S4: The comparison and electron density map of FNPCL between *Dm*AK WT and *Dm*AK H227A. Figure S5: The purification, crystallization and data collection of *Dm*AK H227A. Figure S6: Proposed modalities of structural stability in *Dm*AK H227A.

## Sample Availability

Samples of the compounds are available from the authors.

## Abbrevations

ANS	Anilino-1-naphthalenesulfonic acid
ATP	Adenosine triphosphate
CD	Circular Dichroism
*Dm*AK	*Daphnia magna* Arginine kinase
ECL	Enhanced chemiluminescence
E. coli	Escherichia coli
F	Forward
GdnHCl	Guanidine hydrochloride
IPTG	Isopropyl β-D-1-thiogalactopyranoside
SDS-PAGE	Sodium dodecyl sulfate-polyacrylamide gel electrophoresis
TCA	trichloroacetic acid
PAL	Pohang accelerator laboratory
PCR	Polymerase chain reaction
PDR	phosphate determination reagent
PVDF	polyvinylidene fluoride
R	Reverse
WT	Wild type
