# Supplementary material for: Crystal Structure of H227A Mutant of Arginine Kinase in Daphnia magna Suggests the Importance of Its Stability
Source: Molecules. 2022 Jan 28;27(3):884. doi: 10.3390/molecules27030884 (PMC8839106; doi:10.3390/molecules27030884)
Supplement: Supplementary file 1 [file molecules-27-00884-s001.zip › molecules-1553965-supplementary.pdf]

*Article*

# Crystal Structure of H227A Mutant of Arginine Kinase in *Daphnia magna* Suggests the Importance of Its Stability

Da Som Kim[1†], Kiyoung Jang[2†], Wan Seo Kim[1], Moonhee Ryu[1], Jung Hee Park[1,3]*, Yong Ju Kim[2,3,4]*

[1]*Division of Biotechnology, College of Environmental & Bioresources Sciences, Jeonbuk National University, Iksan 54596, Republic of Korea,*

[2]*Department of Lifestyle Medicine, College of Environmental and Bioresource Sciences, Jeonbuk National University, Iksan 54596, Republic of Korea,*

[3]*Advanced Institute of Environment and Bioscience, College of Environmental & Bioresources Sciences, Jeonbuk National University, Iksan 54596, Republic of Korea,*

[4]*Department of Herbal Medicine Resources, College of Environmental and Bioresource Sciences, Jeonbuk National University, Iksan 54596, Republic of Korea*

*Correspondence: junghee.park@jbnu.ac.kr (JHP); nationface@jbnu.ac.kr (YJK)

[†] Equal contribution in the manuscript

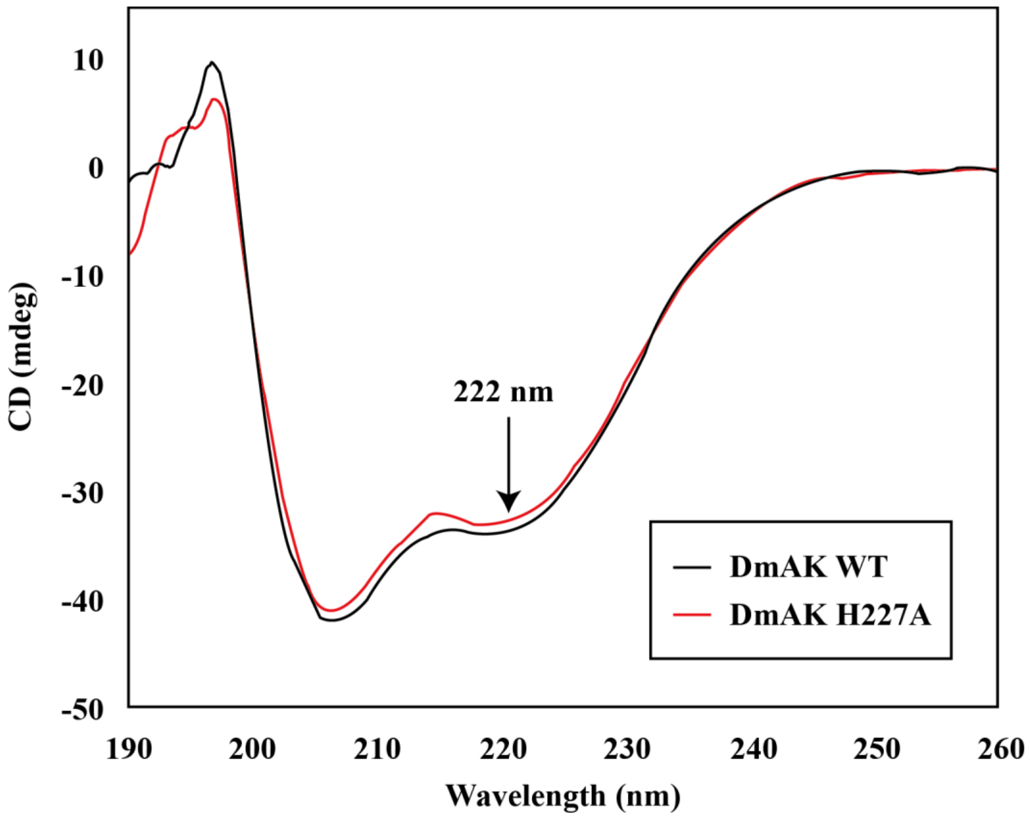


**Figure S1.** CD spectra of *Dm*AK WT (black) and H227A (red). The CD signal was measured from 190 nm to 260 nm.

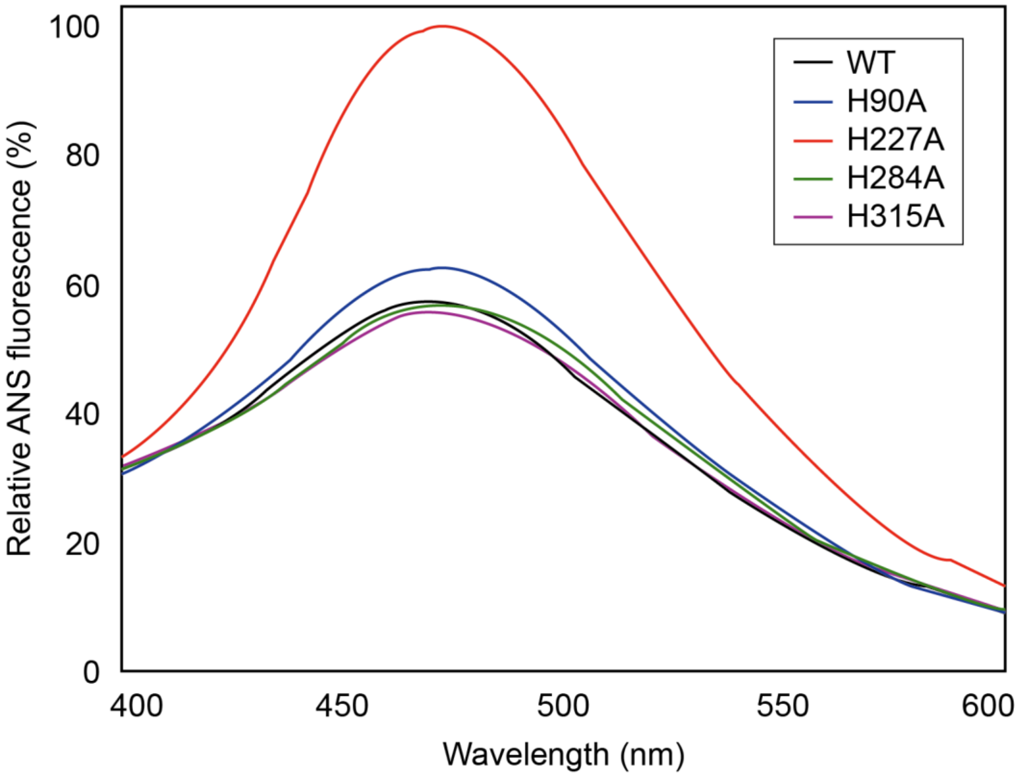


**Figure S2.** Protein unfolding assay of *Dm*AK WT and histidine mutants (H90A, H227A, H284A, H315A) in 0.25 M of Guanidine hydrochloride (GdnHCl) condition. The emission fluorescence data range was from 400 nm to 600 nm by the 380 nm excitation. The data were relatively evaluated by the highest fluorescence of H227A.

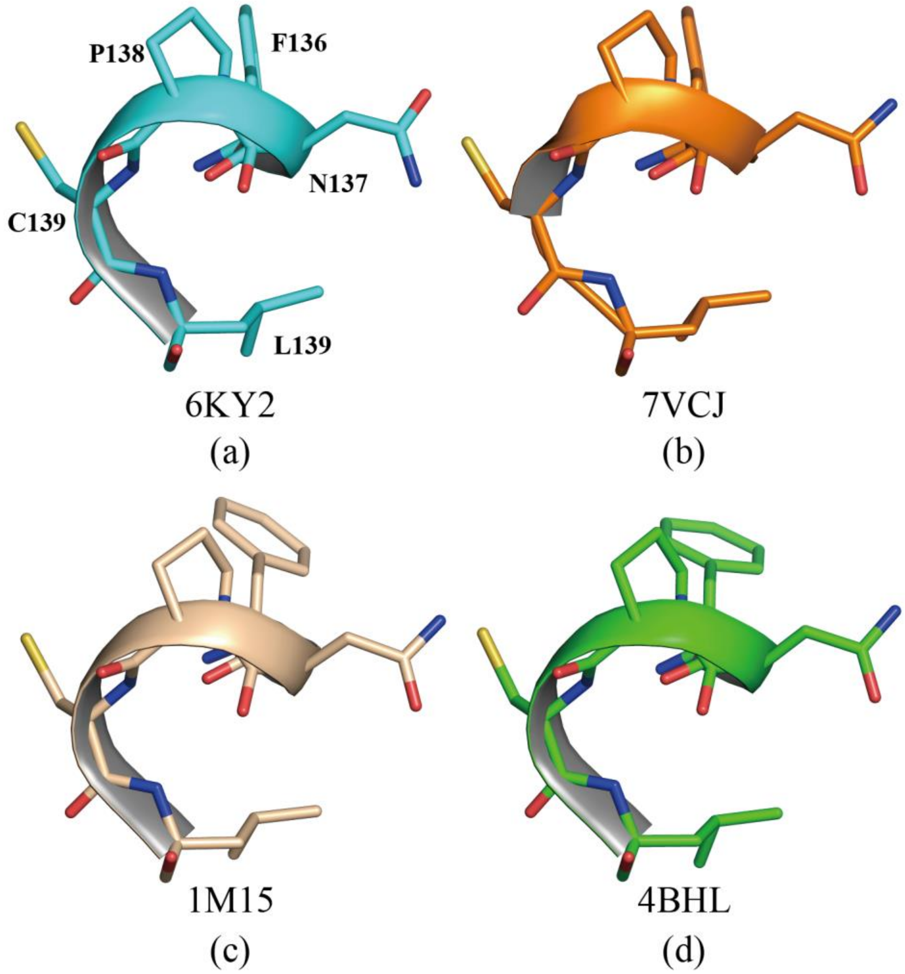


**Figure S3.** The highly conserved FNPCL residues. The FNPCL residues in other AKs are represented by cartoon and sticks model. The secondary structure is formed in *Dm*AK WT ((**a**), PDB entry: 6KY2) but does not be formed in *Dm*AK H227A ((**b**), PDB entry: 7VCJ). Wheat color and green color are FNPCL residues of *Limulus Polyphemus* ((**c**), PDB entry: 1M15) and *Penaeus vannamei* ((**d**), PDB entry: 4BHL), respectively. Secondary structure of FNPCL residues from *Limulus Polyphemus* ((**c**), PDB entry: 1M15) and *Penaeus vannamei* ((**d**), PDB entry: 4BHL) allows the same structure as *Dm*AK WT, while the helix of *Dm*AK H227A is disrupted.

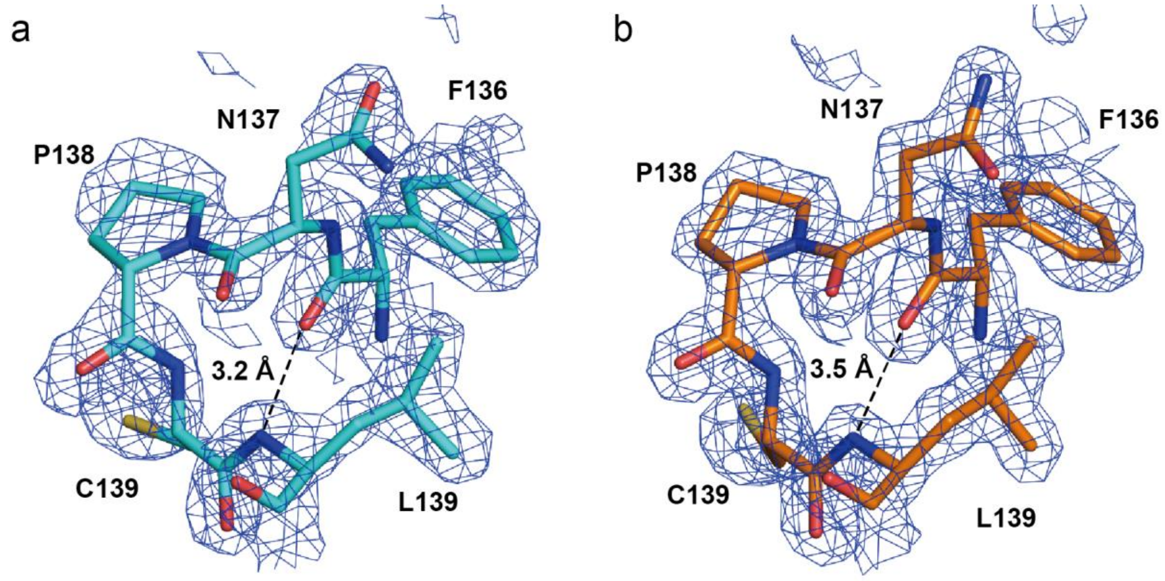


**Figure S4**. The comparison and electron density map of FNPCL between *Dm*AK WT (**a**) and *Dm*AK H227A (**b**). $2F_o - F_c$ electron density map is contoured at 1.5 σ (blue mesh).

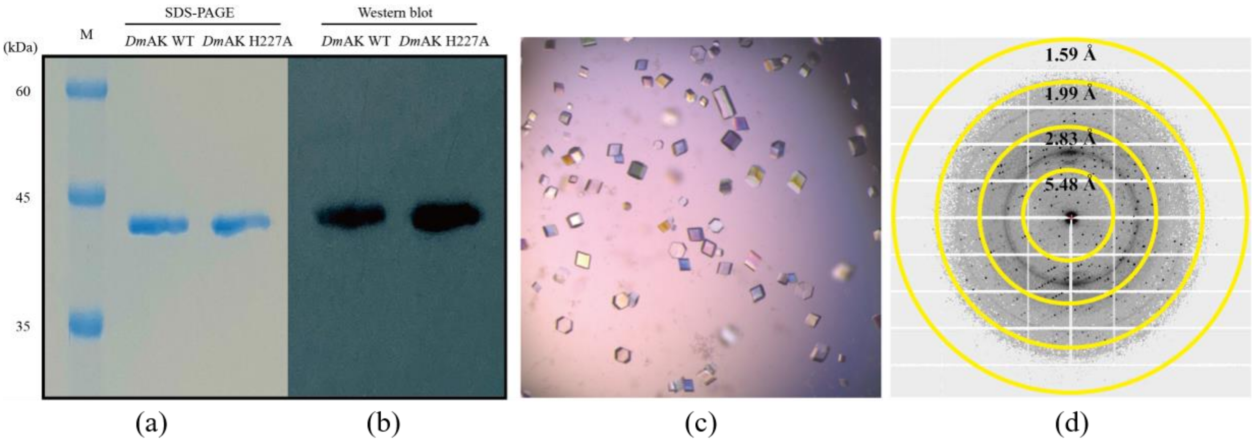


**Figure S5.** The purification, crystallization and data collection of *Dm*AK H227A. The *Dm*AKs (40 kDa) are shown between 35 kDa and 45 kDa on the SDS-PAGE gel (**a**) and in the Western blot (**b**). The purified *Dm*AK H227A was crystallized (**c**) and X-ray diffraction data was collected in PAL 11C (**d**). The resolution rings are indicated by yellow circles.

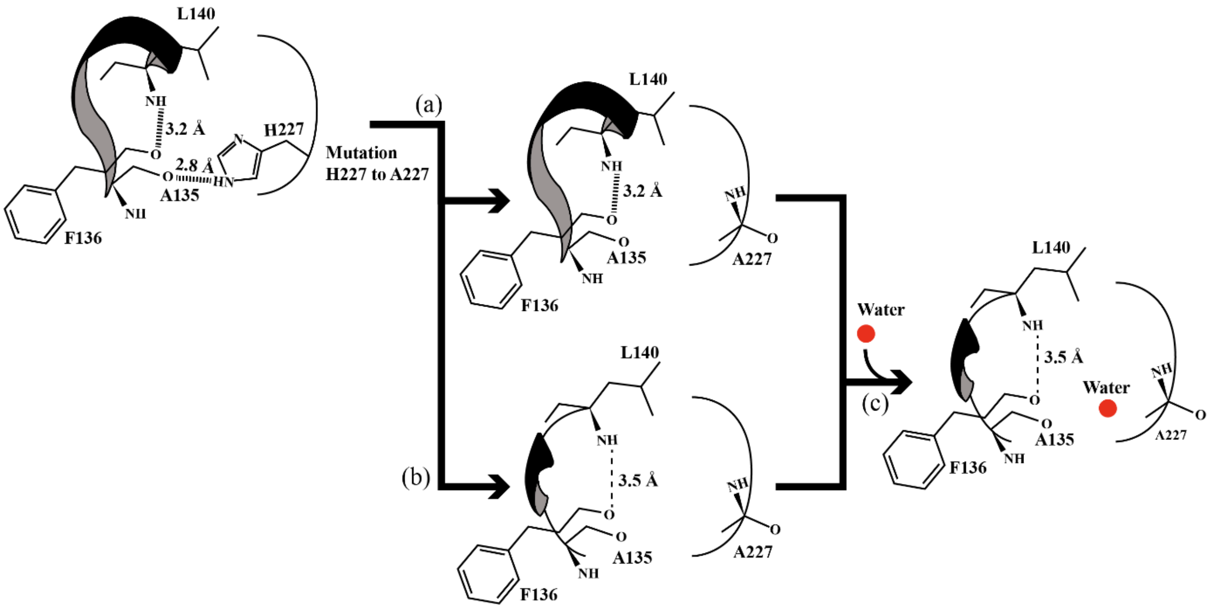


**Figure S6.** Proposed modalities of structural stability in *Dm*AK H227A. First, the mutation of H227 to A227 induce the breakage the H-bond between 135 and 227 residues (**a**) and (**b**). The breakage of these residues might induce the weakness of secondary structure in $^{136}$FNPCL$^{140}$ loop through the increasing distance between F136 and L140 (**b**). Finally, the water molecule is located in site instead of imidazole group of H227 (**c**).
